# The evolution of the United States nucleic acid screening policies

**DOI:** 10.3389/fbioe.2026.1827740

**Published:** 2026-08-17

**Authors:** Gerald L. Epstein, Adeline E. Williams, Curtis Matthew Sharkey

**Affiliations:** RAND Corporation, Arlington, VA, United States

**Keywords:** biosecurity governance, dual use technologies, federal policy development, gene synthesis screening, synthetic biology

## Abstract

**Introduction:**

In 2002, the chemical synthesis of poliovirus demonstrated the dual-use nature of gene synthesis technologies, and private entities initiated the steps towards preventing their misuse. Over the past several decades, nucleic acid sequence and customer screening practices in the United States have emerged from a progression of reactive steps taken by the federal government and private entities.

**Methods:**

This review covers the evolution of nucleic acid synthesis screening guidelines, practices, and potential funding or regulatory requirements in the United States.

**Results:**

In the mid-2000s, private entities developed screening software for voluntary use by gene synthesis companies. Policy analyses subsequently recommended the establishment and use of uniform screening guidelines, and gene vendors formed consortia that acquired or developed screening software. Government guidance on this topic was first released in 2010 and was issued as a voluntary “best practice.” These were codified by the industry as standards, which were updated in 2017. The government revised and finalized their voluntary guidance in 2023, which was enacted, in Executive Order EO 14110, as funding requirements mandating screening. These were countermanded by Executive Order 14292 in mid-2025, leaving the path forward for gene synthesis screening uncertain.

**Discussion:**

Given statutory and regulatory landscapes outlined herein, we discuss the current and potential future state of nucleic acid screening policy.

## Introduction

The nucleic acid sequence and customer screening practices in the United States have emerged from a progression of policy steps taken by private entities and the federal government–working in collaboration, in response to each other, or in response to advances in *de novo* viral synthesis–over the past several decades. These policies are still in development, and their implementation is dependent on public-private partnerships. The entities covered by the emerging policy landscape have expanded in recent years to include nucleic acid synthesis companies as well as nearly all entities involved in the sale, provision, and use of these materials.

## The desire for nucleic acid sequence screening

Industry groups recognized the emerging risks 20 years ago and undertook the first exploration of whether and how companies could ensure that the synthetic genetic materials they were selling would not end up in the hands of people who may misuse them. At least in part, industry’s efforts were intended to ensure that U.S. governmental steps toward screening requirements would be well-reasoned.

During the past decades, both the gene synthesis industry and the federal government have strengthened their practices and recommendations, and each of the past two presidential administrations have indicated their willingness to enforce screening standards. Following through with these intentions, however, has proven difficult. This review covers the evolution of nucleic acid synthesis screening guidelines, practices, and potential funding or regulatory requirements in the United States over the past decades.

## Initial indications of widespread dual-use risks from nucleic acid synthesis

In 2002, Cello, Paul, and Wimmer–scientists from the State University of New York at Stony Brook–published their demonstration that fully synthetic nucleic acids could be used to generate a replicative virus capable of killing human cells in laboratory cell cultures (Cello et al., 2002). The virus also caused paralysis or death in inoculated mice.

The result was not surprising, as more than 20 years prior, in 1981, Nobel laureate Dr. David Baltimore and his then-PhD student Vincent Racaniello had determined the genetic sequence of poliovirus RNA (Racaniello and Baltimore, 1981a). Six months later they showed that DNA constructed using poliovirus RNA as a template, when inserted into mammalian cells, was infectious–it induced the cells to reconstruct the viral RNA, which then caused the cell to produce the full poliovirus particles (Racaniello and Baltimore, 1981b). This was the first infectious DNA clone of an animal RNA virus and served as a proof-of-concept for reconstructing RNA viruses from their genomes (this process is broadly known as reverse genetics).

Even though infectious clones had already been well described by 2002, and it had long been possible to grow large amounts of virus by infecting susceptible cells, the 2002 study shifted the policy discourse because it showed that viruses could be reconstructed using sequencing data and commercially available oligonucleotides, without the need for any live virus as a starting point. A *New York Times* piece entitled “Traces of Terror: The Science; Scientists create a live polio virus” quoted Dr. Wimmer in saying that the study was meant to be a warning that terrorists could make biological weapons “without needing the real thing” (Pollack, 2002).

## Sequence screening and the DNA synthesis industry

### Early approaches to synthesis screening

To minimize the risks associated with synthesizing potentially dangerous DNA, industry began voluntarily screening orders for dangerous sequences. Blue Heron Biotech (now a part of Eurofins Genomics), founded in 1999 by John Mulligan, was one of the first companies to screen DNA orders to identify high-risk sequences that could be misused (Bernauer et al., 2008). For example, DNA encoding a protein toxin could be used to generate a regulated toxin if introduced into cultured cells.^1^ After the 9/11 and anthrax letter attacks in 2001, Blue Heron agreed to serve as a testbed for a software package called Blackwatch–developed by Craic Computing in Seattle. This software compared synthesis orders to databases of sequences from biological select agents and toxins (Bernauer et al., 2008; Tucker, 2010), which are pathogens or toxins designated by the Secretaries of Agriculture or Health and Human Services as posing a severe threat to public health and safety. These are controlled by regulations enforced by the Federal Select Agent Program (FSAP). The genomes of some select agent viruses,^2^ and nucleic acids coding for the production of some select toxins,^3^ are themselves designated as select agents and are thereby also controlled, meaning that government authorization is required of anyone who produces, possesses, transfers, or uses such DNA sequences.

The Blackwatch software matched ordered sequences to a database of select agent genomes, avoiding having to send the sequences to an external database for comparison that might have compromised the confidentiality of the customer order (Bernauer et al., 2008). If an order was flagged as consisting of or being a significant component of select agent pathogen DNA sequences, human follow up was necessary. However, representatives from the DNA synthesis firm Integrated DNA Technologies (IDT) noted that there were not standardized approaches for screening customers and the lack of government regulations about verifying the legitimacy of customers–other than in relation to export controls–complicated the issue of customer screening (Bernauer et al., 2008).^4^

### June 2004: George Church screening proposal

An early conference bringing together synthetic biology practitioners, Synthetic Biology 1.0, was held in June 2004 in Cambridge, MA (Clark, 2004). Shortly afterwards, Harvard synthetic biologist George Church circulated a proposal to mitigate potential risks from the synthesis of pathogens that would be made possible with synthetic nucleic acid synthesis (Church, 2004). The proposal was one of the first to suggest that all synthetic nucleic acid orders be screened for their similarity to known select agents. It also called for the licensing of instruments and reagents and suggested that a DNA Agent Clearinghouse could be established to investigate matches between orders and select agent genomes, look for related orders from other vendors, and contact the vendor and law enforcement authorities in the event of a suspicious order. The proposal also described how such a system could be tested, the provision of exemptions for pre-approved legitimate use of select agent nucleic acids, and the model for covering screening costs.

### December 2004: Passage of “Prevention of Terrorist Access to Destructive Weapons Act of 2004”

On 17 December 2004, Congress passed the Intelligence Reform and Terrorism Prevention Act of 2004 (Public Law 108–458) (Intelligence Reform and Terrorism Prevention, 2004). Section 6906 of this legislation, the Prevention of Terrorist Access to Destructive Weapons Act of 2004, criminalized the unauthorized acquisition, engineering, or synthesis of variola virus, which is responsible for smallpox (Intelligence Reform and Terrorism Prevention, 2004).^5^ Penalties included fines of up to $2,000,000 and imprisonment from 25 years to life. At the time, the construction of variola from synthetic nucleic acids was acknowledged to be very difficult, but the legislation anticipated a future time in which it might become easier. Since the prohibition covered viruses that contained at least 85 percent of the gene sequence of variola virus, which arguably might have included the vaccinia virus that is used as a variola virus vaccine–synthetic DNA manufacturers became concerned that fulfilling a DNA order might unwittingly put them at risk of abetting a very serious felony (Mulligan, 2026).

### May 2006: Synthetic biology 2.0 conference

In May 2006, researchers at the Synthetic Biology 2.0 (SB2.0) meeting in Berkeley, CA discussed how to promote safety and security in the emerging discipline. Attendees included representatives from both academic laboratories and gene synthesis companies and were attending on behalf of both domestic and international institutions (Mulligan, 2006). Some attendees proposed, but meeting attendees as a whole did not formally vote on, a recommendation that DNA synthesis firms screen their orders and report suspicious ones to law enforcement authorities (Service, 2006; Nature, 2006). The meeting also spurred discussion of whether synthesis companies should voluntarily self-regulate or whether formal legislation would be more effective.

### June 2006: The Guardian’s publication of “Did anyone order smallpox?”

In June 2006, the British newspaper *The Guardian* published a story in which the reporter, using an invented company name and the delivery address of a house in London, ordered a 78 base pair (bp) sequence from the 185,000 bp genome of variola (Randerson, 2006a; Randerson, 2006b). The reporter consulted with scientists from Craic Computing and MIT to design a fragment to pose as little hazard as possible (Endy, 2006). In email correspondence with Dr. Drew Endy, the reporter discussed modifying a naturally occurring 120 bp sequence through the addition of 7 mutations to ensure that it would not be translated into potentially hazardous peptides (Endy, 2006), although he ultimately was reported to have ordered a different 78 bp sequence (Randerson, 2006b). The article demonstrated the lack of control over potentially dangerous DNA sequences, noting that many synthesis companies did not, or did not always, screen their synthesis orders. It also highlighted the challenges of screening short (<100) oligonucleotides (oligos), which were reported not to have been screened for at that time, given the risk of false positive hits (Randerson, 2006a; Randerson, 2006b). Most nucleic acid vendors supplied small oligos, as opposed to gene-length sequences, and synthesizing gene-length sequences remained a small portion of the market at that time (National Science Advisory Board for Biosecurity, 2006). The *Guardian* story led to calls for synthesis screening to be more heavily regulated, and it is likely that synthesis firms took it as a warning of the public relations problems, let alone biosecurity problems, which would affect the entire industry if commercially ordered DNA were later found to be used for harm. It should still be noted that some biosecurity professionals have pointed out that ordering short oligonucleotides is only the first step of a complicated process for re-creating a functional pathogen (Jefferson et al., 2014), and that there are many other ways to obtain a pathogen (Gronvall, 2010).

### Summer 2006: formation of the International Consortium for Polynucleotide Synthesis

In the summer of 2006, Blue Heron Biotech and six other companies (GeneArt, Codon Devices, Code Genomics, BaseClear, Bioneer, and IDT) formed the International Consortium for Polynucleotide Synthesis (ICPS) to harmonize biosecurity practices in the industry (Tucker, 2010). The founders sought not only to lessen the potential for the misuse of synthetic DNA, but also to lobby for sensible and well-defined regulation of the gene synthesis market. They wanted to avoid regulations they considered ill-conceived, such as the Prevention of Terrorist Access to Destructive Weapons Act of 2004, and to have a robust regulatory structure in place before anything bad happened.^6^

Importantly, they worked with the FBI to develop a protocol that companies could use to report suspicious ordering activities (Bernauer et al., 2008), but because the consortium relied on volunteer labor, it eventually became inactive (Tucker, 2010). While current guidelines and protocols also include a role for engagement with law enforcement, the nature of the customer-corporate relationship can make reporting to law enforcement a difficult step. Balancing the appropriate reporting of warning signs indicating malign intentions to government authorities with establishing trust among customers and the need to protect their intellectual property is still evolving today.

### December 2006: National Science Advisory Board for Biosecurity report on synthesis of select agents

The National Science Advisory Board for Biosecurity (NSABB) was established in 2004 to provide recommendations to the US government (USG) on biosecurity issues such as the oversight of dual-use research (Holohan, 2005). It formed a Working Group on Synthetic Genomics to assess whether USG policies adequately covered the *de novo* synthesis of select agents, or whether additional oversight was warranted. In December 2006, it approved and released the working group’s report “Addressing Biosecurity Concerns Related to the Synthesis of Select Agents” (National Science Advisory Board for Biosecurity, 2006) that identified existing policies, regulations, and guidance documents most relevant to synthetic select agent genomics:^7^

- The Select Agent Regulations (SAR, 42 CFR Part 73 (Regulations, 2024a), 7 CFR Part 331 (Regulations, 2024b), and 9 CFR Part 121 (Agricultural Bioterrorism Protection Act Regulations, 2024)) control the possession, transfer, or use of nucleic acids that can directly produce infectious forms of a select agent virus and recombinant and/or synthetic nucleic acids that encode for the functional forms of any select agent. Although every select agent virus can be reconstructed from its genome, many of those reconstructions require substantial laboratory manipulation. Only those genomes that are most readily used to construct the corresponding viruses were at that time themselves designated as select agents.
- 18 USC § 175c (U.S. Code, 2004) makes acquisition, engineering, or synthesis of variola virus without government authorization a criminal offense, and Chapter D of Section 175 defines the virus subject to these prohibitions as consisting of smallpox or any derivative of the variola major virus that contains more than 85 percent of the gene sequence of variola major or minor viruses.
- 15 CFR Subtitle B, Chapter VII, Subchapter C (Export Administration Regulations 15, 2024) of the Export Administration Regulations (EAR) governs the export of agents with significant dual use risk, their synthetic genomes, and nucleic acid fragments capable of conferring pathogenicity
- National Institutes of Health (NIH) Guidelines for Research Involving Recombinant or Synthetic Nucleic Acid Molecules (issued in 1976 and renamed in 2013 to explicitly include synthetic nucleic acid molecules) (U.S. Department of Health and Human Services, 2024a) outline biosafety requirements for containment and review of research involving recombinant or synthetic nucleic acids. They call for the establishment of Institutional Biosafety Committees (IBCs) at research institutions to oversee such research.
- Biosafety in Microbiological and Biomedical Laboratories Manual (BMBL), a joint publication of the Centers for Disease Control and Prevention and the National Institutes of Health, provides guidance stating that nucleic acids capable of producing infectious virus or encoding functional select agent toxins should be handled at the same biosafety level as the fully infectious agents or toxins themselves.

After analyzing these documents, the NSABB concluded that additional clarification and more nimble governance options were warranted to keep pace with rapidly evolving science. The authors stated that it would be difficult to develop a suitable regulatory framework because there was a lack of consensus among scientists on how to identify or define sequences associated with select agents (or agents sharing their attributes), or how to best screen orders against such sequences. Nonetheless, they offered the following recommendations:

1. Develop and disseminate harmonized guidance to investigators and nucleic acid providers concerning the select agent regulations with respect to synthetically derived DNA;
2. Develop and mandate common standards for DNA synthesis screening (including ambiguous or partial matches), record keeping, and customer vetting by providers;
3. Tie federal funding to use of compliant vendors;
4. Promote international coordination toward universal screening practices;
5. Repeal 18 USC § 175c (U.S. Code, 2004) because it defines viruses subject to its prohibitions in a way that arguably could capture viruses such as vaccinia (smallpox vaccine) that are not dangerous and have beneficial uses;
6. Clarify and strengthen biosafety guidelines to address synthetic DNA and standardize how “genetic elements” are defined across the Commerce Control List and the select agent regulations; and
7. Convene a group of experts to reassess the select agent regulations, explore a function-based (as opposed to list-based) approach, and consider international implications for synthetic genome oversight.

In response to the 2006 NSABB report, the USG convened an interagency working group on synthetic nucleic acid screening in June 2007 (Tucker, 2010; Eisenstein, 2010). To balance innovation and security for the emerging technology, the interagency working group recommended that the government develop of a set of voluntary guidelines for industry to adopt (Tucker, 2010). Such guidelines could be flexibly updated as science progressed, as opposed to formal legislation that would be more difficult to change. Moreover, voluntary guidance would not necessitate the development of a governmental regulatory infrastructure. This working group ultimately produced the “Screening Framework Guidance for Providers of Synthetic Double-Stranded DNA,” which is discussed below (Eisenstein, 2010; Tucker, 2009).

### October 2007: publication of “Synthetic Genomics: Options for Governance”

In October 2007, authors from the J. Craig Venter Institute (JCVI), the Massachusetts Institute of Technology, and the Center for Strategic and International Studies published the report *Synthetic Genomics: Options for Governance* (Garfinkel et al., 2007). The report assessed the current state of DNA synthesis and identified the technology’s potential risks and benefits. The report made no recommendations but rather formulated a number of policy options aimed at reducing risks of synthetic genomics at three major “intervention points”: (Cello et al., 2002): commercial DNA providers (both gene firms and oligonucleotide manufacturers), (Racaniello and Baltimore, 1981a), owners of benchtop synthesizers, and (Racaniello and Baltimore, 1981b) synthetic DNA users and their associated institutions.

These options include requiring commercial firms to use approved software to screen their orders and requiring those ordering synthetic DNA to be verified as legitimate users by institutional officials. Such policies have been discussed many times during the intervening 2 decades, and they–particularly the first–have garnered widespread support from industry and others. However, although there has been significant movement in this direction in the last few years, they have not yet been implemented.^8^

## Industry consortium guidelines

### The International Association Synthetic Biology’s code of conduct

In April 2008, the International Association Synthetic Biology (IASB), a consortium that had been formed in April of the previous year by five German DNA synthesis firms that were not members of the IPCS held its first industry workshop, “Technical solutions for biosecurity in synthetic biology.” Workshop participants discussed creating a standard Code of Conduct for synthesis screening. The report from this workshop lays out synthesis screening challenges at that time, including the high rate of false positives, standardizing customer screening, and lack of a clear protocol in responding to hits (Bernauer et al., 2008). Later that year, the IASB circulated a draft “Code of Conduct for Best Practices in Gene Synthesis” for comment (Tucker, 2010), and IASB members continued to comment on it through the first half of 2009. The process laid out in the Code of Conduct described automated screening of sequence orders against GenBank, the NIH’s database of all publicly available DNA sequences, with human experts determining the closest GenBank match. If the match was of concern, customer investigation would be conducted before the order was executed. This process was consistent with what many synthesis providers were already doing (Fischer and Maurer, 2010).

However, the manual effort involved in this process proved to be expensive (Maurer and von Engelhardt, 2013), particularly as the price per nucleotide of synthetic nucleic acids was dropping to the point that revenues would not provide much margin to support the cost of this human analytic effort. On the other hand, these verification costs could offset the potential liability of selling nucleic acids to the rare customer who had bad intentions, a tradeoff that remains a major concern in striking an appropriate balance between security and efficiency to this day.

### Competing industry approaches

In July 2009, the IASB announced that it would release the final version of its Code of Conduct in November (Maurer and von Engelhardt, 2013). Shortly afterwards, two of the largest synthetic DNA manufacturers–the German firm GeneArt and the American company DNA2.0, neither of which were IASB members–announced an alternate approach in which nucleic acid orders would undergo an automated screen against a preselected list of concerning sequences that would be continually updated. In what the journal *Nature* characterized as a “standards war,” this approach eliminated human analysis, resulting in its being faster and cheaper, but those advantages came at the cost of not being as comprehensive (Check, 2009). The approach was criticized as not being as effective as the screening methods many firms were already using, and by September, its proponents withdrew it (Tucker, 2010).

On 3 November 2009, the IASB’s Code of Conduct was finalized, and several DNA synthesis companies (including GeneArt but not DNA2.0) met to review it. One major point of discussion was whether companies should try to determine the function of genes their customers were ordering to better clarify the levels of risk, a point not specified in the code. IASB member firms pledged to adopt the code, but other companies at the meeting said that they were still assessing it (Lok, 2009).

### Formation of the International Gene Synthesis Consortium

On 19 November 2009, five non-IASB firms–DNA 2.0 and GeneArt, Blue Heron Biotech, GenScript and IDT–announced formation of the International Gene Synthesis Consortium (IGSC) and the development of its “Harmonized Screening Protocol for Gene Sequence and Customer Screening to Promote Biosecurity.” The protocol included five core components–gene sequence screening, gene customer screening, record keeping, order refusal and reporting, and regulatory compliance (Schubert, 2009).

IGSC’s protocol was very similar to IASB’s; it called for screening every order against comprehensive reference databases, with human expert review required in the event of potential pathogen or toxin orders (Fischer and Maurer, 2010). Specifically, the Harmonized Screening Protocol said that participating companies must compare orders to the IGSC Regulated Pathogen Database (RPD) and to one or more internationally coordinated reference databanks (e.g., GenBank),^9^ translate gene sequences to protein sequences and compare the sequences to protein databases and then use automated screening to identify hits. Positive hits must be reviewed by a human expert and placed in one of three categories: accepted, accepted with further customer review, or rejected. At this time, the IGSC was in the process of curating an RPD and said that in the interim, companies were using their own databases to screen sequences.

Under the IGSC Protocol, customers were asked to provide a shipping address, institution name, country, phone number, and email. Customer names were screened against national databases. Customers purchasing select agent or export-controlled sequences were verified independently and asked to provide the intended use of the sequences. IGSC also asked their member companies to keep written records of sequence and customer screening for 8 years.^10^

## U.S. government guidance

### November 2009: draft U.S. government screening framework guidance

On 27 November 2009, the process triggered by the 2007 formation of the interagency working group on nucleic acid screening resulted in release of HHS’s draft Screening Framework Guidance for Synthetic Double-Stranded DNA Providers (Executive Office of the President, 2009). The proposed guidance had no legal force but was rather offered as voluntary guidance for providers of synthetic double-stranded DNA (dsDNA) that was at least 200 bp in length. The guidance was not directed to vendors of single stranded oligos because select agents and toxins could be much more readily synthesized from dsDNA than from single-stranded (and typically considerably shorter) oligos.

The draft HHS guidance included sequence and customer screening, follow up with customers, and follow up with the U.S. government. It recommended that the company first screen customers to verify their identity, look for “red flags” that might be indicative of an inappropriate end-use, end-user, or destination, and (in the case of foreign orders) check to make sure that the customer was not on any list of prohibited or sanctioned customers. If no such flags turned up, or if they could be resolved with follow-up investigation, vendors were to proceed with sequence screening to determine if an order contained a 200 bp sequence that was more closely related to a regulated microorganism or toxin sequence than to that of any other microorganism (“best match”). Sequences should be screened against the list of biological select agents and toxins or (for international orders) pathogens and toxins listed in the Department of Commerce’s Export Administration Regulations’ Commerce Control List. If sequence screening raised any concerns, the company was advised to follow up and verify the intended use of the sequence. If this follow-up investigation did not resolve those concerns, the guidance recommended that the company seek advice from relevant U.S. governmental departments and agencies by contacting the nearest FBI Field Office Weapons of Mass Destruction (WMD) Coordinator.

The HHS guidance was like the IGSC Harmonized Screening Protocol in recommending customer verification, but it went further to define customer order red flags. On the other hand, whereas the IGSC protocol strictly limited consideration of sequences that may pose biosecurity hazards and should trigger follow up customer verification to sequences from the agents regulated either in the United States or European Union, the HHS guidance did not preclude the use of curated databases of non-biological select agents and toxins or non-Commerce Control List sequences for sequence screening. The HHS guidance acknowledged that synthetic nucleic acids from non-select agents may also pose biosecurity risks and said that companies can choose to investigate such sequences. However, it did not provide additional guidance on what sequences to screen for outside of the biological select agents and toxins and Commerce Control List agents. Those two lists are already regulated by law and specifying them as the targets of screening eliminates the need for firms to make their own determinations as to which sequences pose danger. Moreover, since there are no regulations governing the acquisition or export of non-biological select agent and toxin and non-Commerce Control List microorganisms and toxins, it could be seen as illogical to place constraints on the shipment of DNA from which those microorganisms or toxins might be constructed when no such constraints existed on shipment of the microorganisms or toxins themselves.

To minimize false negatives and to prevent bad actors from circumventing the select agent regulations, the HHS guidance recommended screening both strands of each DNA molecule. For each strand, the guidance recommended screening the nucleotide sequences in both directions (i.e., in the 5’-3’ direction along each of the strands), and also translating the DNA sequence for each strand into corresponding amino acid sequences in each of the three ways in which the sequence can be divided into the 3 bp “codons” that encode individual amino acids. The resulting “six-frame” translations from DNA sequence to amino acid sequence were to also be compared to GenBank’s protein sequences. For both the nucleotide and amino acid sequences, the “best match” was considered as being the segment in the nucleic acid or protein sequence database with the greatest percent identity to the tested sequence. If the best match is to a biological select agent and toxin or Commerce Control List agent and has no equivalent hits to a non-non-regulated biological agent, follow up is recommended.

### November 2009 – January 2010: public review and comment on draft guidance

The November 27 release of the draft guidance triggered a 60-day review period during which comments were formally requested from the public. On 11 January 2010, the American Association for the Advancement of Science hosted a meeting called “Minimizing the Risks of Synthetic DNA: Scientists’ Views on the U.S. Government’s Guidance on Synthetic Genomics” for policy specialists and scientists from academia, the non-profit sector, and industry to discuss the draft guidance (International Gene Synthesis Consortium, 2025). Participants criticized the 200 bp cutoff in the draft HHS guidance and suggested there be no cutoff for screening dsDNA. They also questioned whether limiting screening to select agents was rigorous enough (Tucker, 2010). A summary report from the meeting was submitted as a formal comment to the 2009 Federal Register Notice that had promulgated the draft guidance and solicited comment on it (Executive Office of the President, 2009).

Also, on January 11, the IGSC released a statement that the draft guidance was largely consistent with their Harmonized Screening Protocol, and that they would take “steps to test and implement details of the draft guidance that differ from the protocol” (International Gene Synthesis Consortium, 2025).

### October 2010: issuance of HHS final screening framework guidance

On 12 October 2010, HHS released the final version of the Screening Framework Guidance for Providers of Synthetic Double-Stranded DNA. The Federal Register announcement providing the final text also reviewed the comments that had been received and the corresponding changes to the draft, if any (Executive Office of the President, 2010). Since turning a general intent to screen DNA orders into an operational system to do so requires the specification of many different detailed choices, it is of interest to review the range of public comments about how the government had initially made those choices, and what the government’s response was to these comments. Comments addressed customer screening, customer concerns, and follow-up screening; sequence screening; and other issues:

Customer Issues. Some commenters suggested providing a list of “approved” customers. No change was made because such a list would not be practical; it would require vendors to share their customer lists and would have to be updated too frequently. Additional guidance was requested on how to respond to red flags and whether/how to differentiate between “customers” and “end users.” In response, the final guidance specified that follow-up screening is required whenever a red flag is raised and explained that most initial screening would be focused on customers, whereas follow-up screening would address both customers and “principal users” (a term in the final guidance instead of “end users”). The guidance also provided examples of follow up steps to take, particularly principal users that might be affiliated with large organizations. An option was added to vet unaffiliated customers, or those without publication records, by use of references to verify their identity and the legitimate use of their order. Suggestions to give customers a process to appeal denied orders were not accepted, since companies already reserve the right to deny orders for a wide variety of reasons.

Sequence Screening. The 200 bp threshold for sequence screening was criticized by some as being too short and by others as being arbitrary and not scientifically based. The guidance rejected comments to make the threshold longer and agreed that the 200 bp limit was arbitrary; the final guidance removed the 200 bp threshold and recommended that all dsDNA orders be screened. However, vendors were to make sure that “sequences of concern” (SOC) as short as 200 bp could not be embedded in a longer order without detection. Suggestions to extend screening beyond double-stranded DNA to include oligos, which are single-stranded and typically ordered in lengths considerably shorter than 200 bp, were not accepted because reconstructing agents of concern from oligos is harder than it is than from gene-length dsDNA. Moreover, screening procedures would be significantly harder to implement for oligonucleotide producers than for gene vendors. However, the guidance stated that the USG would continue to monitor this issue as the state of DNA synthesis evolved.

Requests for the government to broaden the definition of SOC, or to provide a SOC database, were not accepted. The select agent and commerce-controlled sequences already to be screened for were the most relevant under current regulations, and it was technically not feasible at the time to develop objective criteria or to compile a database that could be used in a comprehensive and consistent way to identify sequences from other organisms that conferred virulence, pathogenicity, or “other danger.” At the same time, HHS explained that the sequence screening guidance did not preclude use of such a database of sequences that pose biosecurity risks, and that the National Academies was conducting a study on the ability to predict biological function from genetic sequence that might allow such a database to be constructed.

Requests to use a mathematical algorithm for sequence comparison that would require identification of all organisms whose genome exceeded a given degree of similarity (homology) with an order, rather than only the closest or best match, were not accepted. Such an approach would be significantly more human-expert-intensive, and it would also have required clear, effective, and at the time technically infeasible criteria to determine in a non-arbitrary way when follow-up review would be needed. However, minor edits were made in the guidance to clarify technical details.

Other Issues. Providers were also advised to retain records of both customer and sequence screening for 8 years, based on the 8-year statute of limitations for violation of 18 USC § 175b, which prohibits the possession of biological agents that are not in their natural environment and for which there is no reasonable “prophylactic, protective, *bona fide* research, or other peaceful” justification for having them. The 2010 HHS guidance states that it was developed in the context of existing industry protocols and was meant to be implemented without unnecessary costs and to be globally extensible.

### Initial reaction to the guidance

The HHS guidance remained voluntary, even for recipients of federal research funding. Even so, a report from JCVI in October 2015 found that voluntary compliance was pervasive. However, that report also found that “[b]ecause federal grantees and contractors make up a very large percentage of dsDNA customers in the U.S.,” requiring federally-funded researchers to purchase DNA only from providers that complied with sequence and customer screening requirements “would create a strong incentive for companies to comply with the Guidance” (Carter and Friedman, 2015).

Some in the biosecurity community, as well as in industry, embraced the guidance, although the perception of the real risks from access to synthetic DNA differed, and continues to differ, to this day. A commentary by Dr. Gigi Gronvall at Johns Hopkins University titled “HHS Guidance on Synthetic DNA Research is the Right Step” said that the guidance was “adaptable to new technical developments and changing risks, it can be implemented immediately, it can be readily adopted by other countries, and it will cost little” (Gronvall, 2010). However, she went on to say that calls for more stringent oversight should be resisted. For example, developing a database of pathogenic genetic sequences would require significant resources and would likely be too slow to keep up with technical advances in biology. She cited the 2010 National Academies “Sequence-Based Classification of Select Agents: a Brighter Line” (National Research Council, 2010), the report referred to in the HHS announcement of the guidance, which had been released in August of that year (Bhattacharjee, 2010). That study found it was impossible at that time to predict pathogenicity from genetic sequence, precluding the ability to define select agents by their “infectious function.” On the other hand, while many in industry were “relieved” that standardized guidelines were finally established, some contended it was a lower standard than what had already been embraced by industry (Eisenstein, 2010). Finally, others expressed general skepticism that online access to sequences and the decreasing prices of synthetic DNA was significantly increasing the “dual-use threat.” They argued that tacit knowledge–knowledge essential for the successful construction of biological systems that could not readily be codified or transmitted and would therefore not be readily accessible to novices–remained an important barrier to the misuse of biology (Jefferson et al., 2014).

In industry, the IGSC provided protocols to support implementation of the HHS guidance. It responded to the 2010 guidance, in its words, “by publishing the first version of its Harmonized Screening Protocol [v2] – a document intended to help IGSC members design and implement screening programs. The IGSC also created its Restricted Pathogen Database (RPD), a data set used by member companies for sequence screening” (Harmonized Screening Protocol v3, 2024). Indeed, the IGSC Harmonized Screening Protocol v2 published in 2017 reads that it is meant to “help ensure broad compliance with HHS Guidance for Double Stranded DNA Providers and other international standards” (Harmonized Screening Protocol v2.0, 2021).

## Modernization of the IGSC harmonized protocol

On 19 November 2017, 8 years after publishing its initial Harmonized Protocol, the IGSC released an updated version, Harmonized Protocol [v2] (Harmonized Screening Protocol v2.0, 2021). By this time, the IGSC had deployed its common RPD that IGSC members used to screen sequences, in addition to one or more other internationally coordinated sequence reference databanks (e.g., GenBank). The RPD includes data from select agents, the Australia Group list, and other national lists of regulated pathogens. The Harmonized Protocol 2.0 established that the RPD must be updated annually to include all gene sequences identified as potentially hazardous by authoritative bodies.

The updated protocol was also clearer in customer screening guidelines. IGSC members commit only to supply DNA sequences from regulated pathogens or toxins to researchers in “*bona fide* government laboratories, universities, non-profit research institutions, or industrial laboratories demonstrably engaged in legitimate research.” The term *bona fide* was later incorporated into the 2023 revised guidance as a requirement to purchase SOCs.

Compared to the 2010 US guidance, the 2.0 Protocol was not limited to screening for select agents, allows for more flexible screening methods other than the Best Match approach, and incorporates human review and interpretation for ambiguous hits. The IGSC continued to update its screening protocol. The Harmonized Protocol 2.1, which was released on 5 May 2020, added a recommendation to screen oligonucleotide pools, or mixtures containing a great many different oligonucleotide sequences (Harmonized Screening Protocol v3, 2024).

## Addressing evolving capabilities and newly recognized risks

### Synthesis of horsepox virus

On 19 January 2018, Canadian virologist David Evans and colleagues demonstrated, and published a protocol for, the construction of horsepox virus in cultured cells using only synthetic nucleic acids (Noyce et al., 2018). As the synthesis of poliovirus more than a decade earlier had shown, viruses that have genomes consisting of positive sense single stranded RNA (+ssRNA) can fairly readily be constructed with synthetic nucleic acids.^11^ Many regulated viruses are + ssRNA viruses, and protocols to reconstruct these and most other viruses from their genomes are available. However, the generation of synthetic orthopox viruses had been considered infeasible due to their very large double-stranded DNA genomes and the difficulty of producing certain hairpin-like features on their genome (Noyce et al., 2018). Evans’ protocol changed this. It was one that many molecular biologists would be able to carry out (DiEuliis et al., 2017). Although horsepox is not a regulated virus, it is in the same genus (*Orthopoxvirus*) as variola, the virus responsible for smallpox. Given the massive risks posed to all societies from the reintroduction of variola, which would likely be amenable to generation through the same strategy, both the federal government and the gene synthesis community began considering how screening practices could be strengthened and harmonized to address the potential for harm associated with emerging capabilities.

### Additional screening approaches

Shortly thereafter, in 2019, the MIT Media Lab launched the Secure DNA Project, an effort to develop synthesis screening technology that was meant to minimize the number of false positives and fully automate the process (Secure DNA, 2026). The system breaks orders into fragments and directly compares them to a database of random sequences from potential bioweapon sequences (Secure DNA, 2026). Importantly, both customer orders and the sequence database are encrypted and they are compared in encrypted form. This process assures customers that their intellectual property (as reflected in their DNA orders) is secure and also prevents the database from being mined to yield a roadmap of dangerous sequences. As of April 2026, the software continues to be freely available (Secure DNA, 2026).

After the World Economic Forum and the Nuclear Threat Initiative (NTI) called for an international Common Mechanism for DNA synthesis screening in 2020, the International Biosecurity and Biosafety Initiative for Science (IBBIS) was launched in 2024. IBBIS has developed such a mechanism, which provides free, open-source, screening software (Crawford et al., 2024).

### Revision of the 2010 screening framework guidance

The Federal Register notice accompanying the release of the 2010 HHS Screening Framework Guidance stated that it would be “reviewed on a regular basis and revised, as necessary” (Executive Office of the President, 2009). Since the release of that guidance, DNA synthesis technology continued to evolve rapidly, and synthetic nucleic acids were being increasingly used in life science research and biotechnological applications. Federal interagency meetings had been convened to discuss updating the guidance, such as a meeting with representatives from JCVI to discuss the findings in their 2015 report,^12^ but no concerted effort was mounted by the U.S. government to review the guidance until 2018, when the White House Office of Science and Technology Policy initiated a process to do so. That review was to take into account how the relevant technologies, commercial markets, and security threats involving the misuse of synthetic DNA had evolved since 2010, and to propose revisions to the guidance to account for those changes. HHS convened several meetings on this topic in 2018, but those initial steps did not proceed into a formal review, and the review process went into abeyance. The process restarted on 26 August 2020, when the HHS Assistant Secretary for Preparedness and Response (ASPR) published a request for information (RFI) in the Federal Register on whether and how the 2010 guidance should be modified (U.S. Department of Health and Human Services, 2020). The RFI contained motivating questions in the following topic areas: scope of the guidance, sequence screening, biosecurity measures, customer screening, minimizing burden of the guidance, technologies subject to the guidance, and additional considerations. Samples of the 25 questions in the RFI included:

- Should the focus extend beyond select agents and the Commerce Control List?
- Should the scope extend beyond dsDNA?
- Should the guidance be updated to identify SOCs?
- Are there other alignment approaches other than Best Match?
- Are there alternative security or screening approaches to the screening mechanism that could be established?
- Are there additional end-user screens or follow-up mechanisms that should be considered?
- Does the guidance cause any undue burden?

The full comments received in response to the 2020 RFI, along with a summary of those comments by topic, were published online by HHS (U.S. Department of Health and Human Services, 2021a; U.S. Department of Health and Human Services, 2021b). Both the responses to the open-ended questions in the RFI and the issues raised in responses to each of the RFI’s categories reflect experience that had been gained in applying the 2010 guidance as well as the evolution in thinking about screening since that guidance was released. These comments are summarized in Table 1.

**TABLE 1:** A summary of the central themes raised by stakeholders in feedback to the 2020 HHS RFI about potential revisions to the 2010 HHS guidance.

Scope	• General agreement about extending the scope beyond biological select agents and toxins and Commerce Control List agents (i.e., regulated agents) • Broad consensus found in stakeholder comments that the guidance should be expanded beyond providers to other entities in the nucleic acid lifecycle (e.g., entities transferring nucleic acids beyond point of sale) • Concerns that such an expansion could lead to downsides, such as increasing the cost of screening for compliant providers, as well as about burdens on life sciences researchers • Challenges associated with defining which sequences are SOCs beyond those from regulated agents • The complexity of establishing lists of SOCs and of continuously curating them in light of new discoveries about mechanisms of pathogenesis
Sequence screening methodology	• Broad consensus found in stakeholder comments suggesting reducing the screening window to 40–50 bp from the 200 bp window in the previous HHS guidance • Respondents generally favored retaining the suggestion for using BLAST’s best match threshold for matching orders to SOCs • Some respondents noted that the federal government could provide performance standards against which alternative approaches could be evaluated • Stakeholders stressed that the lack of a list maintained and promulgated by the federal government had created inconsistencies in the implementation of the government’s guidance • Stakeholders raised that individual orders should be assessed in combination with other orders from individual customers to determine whether smaller fragments than the screening window from multiple individual orders could be assembled into longer nucleic acid fragments that themselves would constitute SOCs
Biosecurity measures	• Respondents indicated that it was possible and would be helpful for the government to make available a database of SOCs, with sequences listed based on pathogenic functions • Implementation was impeded by the lack of biosecurity experts who could evaluate the results of sequence and customer screening • HHS could help overcome the lack of biosecurity expertise by investing in workforce training
Customer screening	• Respondents recommended measures to streamline customer screening, including pre-screening, white-listing, and the maintenance of a restricted persons blacklist, as potential U.S. government responsibilities
Minimizing burden	• Stakeholders noted that the cost of screening had increased markedly as a share of the overall cost of oligonucleotide synthesis, given the need for expensive expert staff for follow up as compared to the steadily dropping cost for synthesizing the nucleic acids themselves • Stakeholders noted that one of the best ways that the federal government could reduce screening costs would be to provide an annotated database of sequences against which to screen • Respondents commented that clarification about what specific types of data about customers and sequences needed to be retained and the required latency for data retrieval would significantly alleviate the burden of compliance • Respondents noted that the burden associated with complying would be alleviated by the federal government providing a mechanism – such as an application programming interface (API) – to support the uniform appreciation of: oGenes recognized as containing SOCs; and owhitelisted customers
Technologies subject to the Guidance	• There was broad consensus that screening should be extended to each type of synthetic oligonucleotide, not just dsDNA. • Some responses indicated that benchtop DNA synthesizers posed a serious biosecurity threat

## Final updated screening guidance for the United States

On 29 April 2022, ASPR published a draft revised screening framework guidance as a Notice in the Federal Register (87 FR 25495), which represented a significant expansion from the original 2010 guidance. For example, in addition to covering synthetic dsDNA providers, the revised guidance covered institutions, principal users, end users, third-party vendors of oligonucleotides, and synthesis equipment providers. In contrast to only covering dsDNA, the revised guidance includes “DNA or RNA, single- or double-stranded, of lengths 50 bp or longer if ordered in quantities of less than 1 μmol, or lengths 20 bp or longer if ordered in quantities of 1 μmol or greater.” The revised guidance also defines SOCs as “sequences that contribute to toxicity or pathogenicity, whether derived from or encoding regulated or unregulated biological agents,” extending the scope beyond pathogens in the select agent regulations and Commerce Control List. A footnote defines pathogenicity or toxicity as threatening “public health, agriculture, plants, animals, or the environment. SOCs include sequences for which a direct and harmful impact on a host has been verified based on published experimental data; and, where experimental data do not exist, based on homology to a sequence encoding a verified function.”

Comments received in response to the Federal Register Notice publication of the draft revised guidance were published on an HHS website and were thoroughly reviewed (U.S. Department of Health and Human Services, 2022; Sharkey et al., 2024). Many comments were complimentary of the expanded scope in the revised Guidelines, particularly of reducing the screening window from 200 to 50 bp, including nucleic acids other than dsDNA, and expanding beyond agents on the biological select agent and toxin list and the Commerce Control List. They also praised the addition of a periodic review to ensure guidance evolves with technological advances.

Much of the criticism found in the public comments focused on the SOC definition, which several commenters found to be ambiguous. There was broad support for curating a centralized database of SOCs, although some flagged that making that database public in the interest of transparency could create information hazards. Several commenters also stressed the need for clear guidelines, education, and resources to support determinations of customer legitimacy.

Although many agreed benchtop synthesizer manufacturers should be covered, discussion focused on how screening could be done once the machines were in the hands of users. Several noted that the manufacturer would need to track the equipment throughout its lifecycle, and that the Guidance did not provide recommendations on this front.

Finally, many highlighted the increased costs associated with compliance, including building, implementing, and maintaining state-of-the-art screening systems, investigating false positives, and enhancing customer screening. They emphasized that the USG should support developing tools, standards, and best practices.

After reviewing comments, HHS issued a final guidance in October 2023 (U.S. Department of Health and Human Services, Administration for Strategic Preparedness and Response, 2023). Table 2 summarizes the substantive changes in the HHS screening framework guidance between the 2010 and 2023 versions.

**TABLE 2:** Comparison of critical elements in iterations of HHS nucleic acid synthesis screening frameworks from 2010 and 2023.

Feature	2010 Guidance	2023 Final guidance
Scope	dsDNA with 200 nt sequence screening window	dsDNA, ssDNA, dsRNA, ssRNA ≥50 nt in length, with the recommendation that entities consider the possibility that multiple synthetic orders of short nucleic acids could be assembled into SOCs
Agents covered	SAR and CCL	SAR and CCL, plus SOCs found in regulated or unregulated organisms that threaten public health agriculture, plants, animals, animal products, or the environment
Who is responsible	Synthetic dsDNA providers	Synthetic nucleic acid providers plus research institutions, principal users, end users, third-party vendors, and synthesis equipment manufacturers
Customer vetting	Verify customer identity for all orders and look for ‘red flags’ that may indicate misuse	Verify the identity of all customers, verify the legitimacy of customers ordering materials containing SOCs, verify legitimacy of customers ordering SOCs, and look for “red flags” that may indicate misuse – with examples of how to verify legitimacy provided in the policy and its companion Guide (administration for strategic preparedness and response. Companion Guide to assist in implementing the recommendations of the screening framework Guidance for providers and users of synthetic nucleic acids. U.S. Department of health and human services. October 2023. Available from: https://aspr.hhs.gov/S3/Documents/SynNA-Companion-Guide-508.pdf)
Sequence screening	Best match to GenBank	Best match, with attention also given to improved tools/databases beyond BLAST and best match
Record keeping	Retain customer and sequencing screen records for 8 years	Specifies what information in orders containing SOCs to archive for 3 years (or up to 8 years if possible), as well as customer orders, protocols, screening documentation, and follow up; also to maintain records of all transfers involving SOCs

BLAST is basic local alignment search tool, CCL is the Commerce Control List, ds is double-stranded DNA, nt is nucleotides (corresponding to base pairs in double-stranded nucleic acids), SAR is select agent regulations, SOCs are sequences of concern, ss is single-stranded.

The final 2023 HHS Screening Framework Guidance for Providers and Users of Synthetic Nucleic Acids (88 FR 70998) (U.S. Department of Health and Human Services, Administration for Strategic Preparedness and Response, 2023) was intended to assist all entities involved in synthetic nucleic acid synthesis develop and operate effective screening procedures, including the detection of orders meant to circumvent screening. It recommended that customers self-identify when their orders contain SOCs and preemptively provide evidence of their legitimacy, that providers and third-party vendors perform sequence screening, verify customer identities, and verify legitimacy when orders containing SOCs are placed; and that all stakeholders keep record of orders with SOCs as well as any and all transfers. It calls for benchtop synthesizer manufacturers to distribute equipment capable of synthesizing SOCs only to customers proven to be legitimate, and that the companies track legitimate use of the machines. If SOCs are identified during sequence screening, providers must follow up with the customer, and providers can facilitate follow-up by providing a mechanism for users to preemptively self-report information to establish their legitimacy when they are aware that their orders contain SOCs. The guidance also emphasizes that pre-existing practices can be used (and potentially adapted) to verify legitimacy and track transfers.

This final guidance also elaborated on how benchtop synthesizer manufacturers could develop best practices, including screening all customers, verifying legitimacy, tracking use, incorporating SOC screening into the machines, and including data logging.

In the Sequence Screening Methodology Section, the guidance acknowledged that a “database of known sequences that contribute to pathogenicity and toxicity in humans, animals, and plants, and that have a direct and harmful impact on a host, may not yet exist or may not yet be fully developed.” The guidance encouraged “industry consortia and/or any other interested parties in the continued development of such a database for screening SOCs—provided that measures are taken to prevent such a database from being misused.” It ends by encouraging new methods, including predictive ones, to identify SOCs.

HHS also published an accessory document, the Companion Guide to Assist in Implementing the Recommendations of the Screening Framework Guidance, which provides example scenarios and additional information on how to carry out the recommendation in the guidance (U.S. Department of Health and Human Services, 2023). It suggests that providers, including benchtop synthesizer manufacturers, have well documented sequence screening protocols, share best practices, and periodically evaluate screening effectiveness. Ways to recognize cybersecurity vulnerabilities–an issue raised by commenters to the draft of the guidance–are also discussed.

The Companion Guide provides clear indicators to verify customer legitimacy, as well as their appropriateness to use the SOCs they have ordered. It also provides seven use cases that demonstrate how providers should respond when receiving orders containing SOCs from different types of customers. It identifies red flags that may arise while verifying customer legitimacy that should result in follow-up, as well as indicators to help identify suspicious orders, such as unclear identities, inconsistent proposed uses with job title, and unusual methods of payment or shipping procedures. It also provides example scenarios of when to contact the nearest FBI Field Office’s WMD Coordinator or with the Department of Commerce (DOC).

Finally, the Companion Guide elaborates on compliance obligations under the Export Administration Regulations (EAR). Those regulations control the export and transfer (including deemed exports to foreign nationals within the United States) of dual-use genetic elements and organisms linked to pathogens and toxins on the Commerce Control List, requiring providers of synthetic nucleic acids to consult these rules and obtain licenses when necessary.

### State-level screening requirements

Two states, Maryland and California, have undertaken legislative approaches to require gene synthesis screening for researchers at institutions that receive state funding. Whereas requirements were established in California in 2022, efforts in Maryland to establish screening requirements stalled in 2021.

On 8 February 2021, Maryland House Bill (HB) 1,256 was introduced, requiring the Maryland Department of Health and Mental Hygiene to develop gene sequence and customer screening guidelines, along with a certification and compliance review mechanism for gene synthesis providers and manufacturers, by 2023 (Assembly, Maryland General, 2020). Providers operating without certification would face civil penalties up to $1,000 per day. Entities receiving State resources would be required to purchase synthetic nucleic acids exclusively from certified providers. Testimony in favor was presented by Dr. Gigi Gronvall of the Johns Hopkins Center for Health Security (Gronvall, 2021) and in opposition by Pam Kasemeyer, representing the Maryland Tech Council (Kasemeyer and Kaufmann, 2021). Their opposition included the point that the “matter requires federal regulation, rather than a patchwork of state laws and regulations that may differ state by state and may not align with federal regulations.” The bill was withdrawn on 5 April 2021, before a floor vote.

In California, Assembly Bill (AB) 1963, signed into law on 26 August 2022, requires the California State University system (and requests the University of California system) to develop systemwide purchasing guidance for gene synthesis products and equipment (California State Assembly, 2022). The guidance must ensure purchases are made from providers that implement safeguards against misuse of synthetic genes, and it directs the universities to consider adopting the IGSC screening criteria in their standards.

In comparison to Maryland’s withdrawn HB1256, which sought to create state-level regulatory requirements for gene synthesis vendors as well as to impose screening requirements on DNA purchased with state funds, AB1963 required some state universities to adopt procurement guidance ensuring synthetic nucleic acids were purchased from providers that followed biosecurity safeguards. Both state bills embodied the recommendations from the 2015 JCVI report suggesting that funding requirements be established for procuring only from providers that diligently implement screening methodologies (Carter and Friedman, 2015).

## From guidance to funding requirements–presidential action

On 30 October 2023, President Biden signed Executive Order (EO) 14,110, *Safe, Secure, and Trustworthy Development and Use of Artificial Intelligence* (Executive Office of the President, 2023b). Section 4.4(b)(i) of the EO called out the intersection of artificial intelligence and biotechnology as a pressing security risk and, to reduce the potential misuse of synthetic nucleic acids, directed the Office of Science and Technology Policy (OSTP) to establish a framework “encourag[ing] providers of synthetic nucleic acid sequences to implement comprehensive, scalable, and verifiable” procurement screening mechanisms such as those described in the 2023 HHS guidance (U.S. Department of Health and Human Services, Administration for Strategic Preparedness and Response, 2023). The EO also tasked the DOC’s National Institute of Standards and Technology (NIST) to work with industry in defining technical specifications, best practices, and conformity assessment for such screening; required federal funding agencies to mandate use of framework-compliant providers as a condition of grant support; and directed the Department of Homeland Security (DHS) to develop a stress testing and evaluation framework, with annual reports recommending improvements to nucleic acid procurement screening systems.

In response to the EO, the National Science and Technology Council (NSTC) established the Fast Track Action Committee on Synthetic Nucleic Acid Procurement Screening, which published the final version of its *Framework for Nucleic Acid Synthesis Screening* in September 2024 (National Science and Technology Council, 2024). The NSTC framework established requirements, to take effect 26 April 2025, that all federally funded researchers purchase synthetic nucleic acids through providers and manufacturers that adhered to the framework. Manufacturers and providers were to:

1. Provide documentation, either on their website or when requested, that they are adhering to the screening framework
2. Screen for SOCs in purchase orders
3. Screen customers purchasing SOCs or benchtop synthesizers to verify legitimacy
4. Report potentially illegitimate purchases^13^
5. Retain records of synthetic nucleic acid or benchtop synthesizer purchases for at least 3 years^14^ ^,^ ^15^
6. Ensure cyber- and information security

The NSTC framework expanded the set of covered providers to include biofoundries, cloud labs, core facilities, and contract research organizations (CROs). It also added the following practices for SOC screening to be implemented in 2026: (Cello et al., 2002): screen 50 nucleotide (nt) windows for SOCs, (Racaniello and Baltimore, 1981a), apply screening methods that detect the potential for short nucleic acid sequences to be assembled into longer SOCs for bulk or multiple orders, and (Racaniello and Baltimore, 1981b) make efforts to screen additional SOCs known to contribute to pathogenicity or toxicity in non-regulated agents. Acknowledging that the state of science would likely change by 2026, the OSTP framework defined three criteria to inform SOC designation:

- Scientific evidence showing the sequence contributes to pathogenicity or toxicity in humans and animals
- Degree to which sequence is likely to be recognized as a candidate for misuse
- Ease with which sequence could be misused

The draft NSTC framework published in April 2024 covered any sequences that are uniquely found in a regulated agent. However, in response to feedback from stakeholders (Godbold et al., 2025), the final framework released the following September limited its purview to genes from regulated agents that are known to contribute to pathogenicity or toxicity. This shift represented a significant break from the approach taken in the U.S. synthesis screening policies published during the previous decade. Whereas U.S. export controls have long been limited to the regulation of genes from listed agents that “[c]ould endow or enhance pathogenicity” (Part 774, 2026), both the 2010 and the 2023 HHS screening framework guidance documents had included all sequences unique to regulated agents. The September 2024 shift to regulating only sequences known to contribute to pathogenicity or toxicity represented a novel direction for federal guidelines to synthetic nucleic acid providers.

Verifying customer legitimacy, record keeping, and cybersecurity requirements were largely the same in the 2024 NSTC Framework as had been outlined in the 2023 HHS guidance. Building on the 2023 guidance that recommended customers self-report SOC purchases, the NSTC framework recommends providers include a mechanism on their ordering system where customers can so specify.

The NSTC framework also established the first requirement for sequence screening, mandating that federal-funded researchers purchase synthetic nucleic acids only from providers that self-attest to meeting the requirements of the framework. The framework did not exhaustively outline how compliance would be measured or verified, although EO 14110 had already tasked NIST with developing and refining the following for use by synthetic nucleic acid sequence providers:

- “Specifications for effective nucleic acid synthesis procurement screening;
- Best practices, including security and access controls, for managing sequence-of-concern databases to support such screening;
- Technical implementation guides for effective screening; and
- Conformity-assessment best practices and mechanisms.” (Executive Office of the President, 2023a)

It also tasked the DHS with developing the structured evaluation and stress testing mechanisms called for in Executive Order 14110. However, neither the specifications, best practices, technical implementation guides, or conformity assessment materials tasked to NIST or the evaluation and stress testing methodologies tasked to DHS were produced by those agencies prior to the Trump Administration’s 23 January 2025 rescission of the Biden Administration Executive Order that had called for them (Executive Office of the President, 2025b).

## Updates to industry consortia (IGSC) requirements

On 3 September 2024, after the publishing of the OSTP Screening Framework, the IGSC published a v3.0 of their Harmonized Screening Protocol to provide “specificity and context” for synthesis screening systems and to better align with the 2023 revised HHS guidance, EO 14110, and the OSTP Framework (Harmonized Screening Protocol v3, 2024). It points out that although no company is legally required to screen sequences, some sequences are regulated under FSAP or export controls, leaving the provider liable should it supply those sequences to customers not authorized to receive them. It lists relevant domestic- or export controls (e.g., the FSAP or EAR, respectively) as well as applicable government guidance and screening data resources to support its members.

The v3.0 provides recommendations on how to verify legitimacy of SOC orders that include ensuring the customer comes across as knowledgeable and consistent, and it suggests that the provider have sufficient expertise to understand the detail of the research. It lays out customer screening, sequence screening, record keeping and retention, order refusal and reporting, and regulatory compliance that well mirror the guidance documents previously discussed. Some noteworthy details are: IGSC members must retain records of every gene sequence screening result for at least 8 years (although the HHS guidance recommends 8, it only requires 3) and that IGSC members do not sell or ship DNA or fragments of regulated pathogens to distributors or resellers unless they identify end users and demonstrate their compliance.

## International partners in biosecurity policy

The United Kingdom screening guidance on synthetic nucleic acids for users and providers, published 8 October 2024 by the Department of Science, Innovation, and Technology (DSIT), is similar to the US guidance and is for all users and providers (including third-party vendors and benchtop synthesizer manufacturers) in the United Kingdom (Department for Science, Innovation and Technology, 2024). SOCs are defined as those that contribute to pathogenicity, virulence (not mentioned in the US definition), or toxicity and are a best match to current and future iterations of UK-regulated agents except when also in an unregulated organism or toxin.

The general synthesis screening workflow is like that of the US, although there are some notable differences. Like in the US screening frameworks (U.S. Department of Health and Human Services, Administration for Strategic Preparedness and Response, 2023; National Science and Technology Council, 2024), customers are instructed to self-identify when their orders contain SOCs and to preemptively provide information to providers that establishes their legitimate use for the materials they are ordering, while providers must verify customer identity for every order. The United Kingdom guidance further notes that providers must retain customer information for an unspecified amount of time and refer to it for repeat orders; every 18 months, providers must ask customers to update their information. Depending on what regulated organism an SOC is from, the provider must ask the user to submit proof they are legally authorized to obtain the sequence. A user is also required to perform a risk assessment on any synthetic sequence that could be considered a “harmful substance” by United Kingdom law. Finally, the user, as well as the provider, must retain records of SOC orders and transfers for at least 3 years.

The EU Biotech Act, introduced in December 2025 and currently under consideration, includes regulatory language for “biotechnology products of concern,” which includes both synthetic nucleic acids encoding SOCs as well as benchtop nucleic acid synthesizers. Whereas sequence screening policies in the United States have been limited to guidelines or potential requirements for governmental funding recipients, the Act would establish a regulatory requirement to screen nucleic acid synthesis orders and verify the legitimacy of customers ordering them or benchtop synthesizers (European Commission, 2025).

Subsequent to 2023s EO 14110, NIST supported the development of nucleic acid synthesis standards by the International Organization for Standardization (ISO). NIST led the U.S. Technical Advisory Group to ISO’s Technical Committee 276 Working Group 6, which was focused on nucleic acid- and protein-based devices (National Institute of Standards and Technology, 2025). This working group issued an international standard for quality control in the synthesis of double-stranded DNA in 2024 (ISO, 20688-2, 2024), which includes standards for screening synthetic DNA orders for sequences of 50 bp or greater that either encode for harmful biological functions or directly endow or enhance toxicity or pathogenicity. Although ISO 20688–2:2024 is limited in its scope to double stranded DNA orders, its codification of the expanded SOC definition along with its requirement that customers be identified, and that the information about them is consistent with the materials that they are ordering, is a significant step toward cementing the intent of the 2023 and 2024 U.S. government policies in the international marketplace for synthetic genetic materials.

## There will be a test

After the 2023 HHS guidance was issued, and amid the development of the 2024 White House policy that would have established screening as a requirement of federal research funding, a series of publications investigated the effectiveness of customer and sequence screening by companies selling nucleic acids. For example, in May 2024, Edison, Toner, and Esvelt found that 36 of 38 companies tested were willing to provide gene-length DNA sequences that were not identical to sequences from any regulated pathogens, but from which the 1918 influenza virus genomic sequences could be reconstructed with relatively simple laboratory manipulations. Since 1918 influenza virus is a select agent, unobscured orders for its genome should be flagged under current screening procedures and guidance. Twelve of these companies were IGSC members (Esvelt, 2024; Edison et al., 2024; Edison et al., 2026). It is worth noting that shortly thereafter, IGSC responded that the study was misleading because the companies from which the synthetic genetic materials were ordered were aware of the affiliation of the orders with prominent laboratories at the Massachusetts Institute of Technology, and its member companies had correctly performed sequence and customer screening (IGSC, 2024). Around that same time, Kane and Parker surveyed the “screening state of play” and interviewed 18 nucleic acid screening companies to determine strengths and gaps in what was then current DNA provider security practices (Kane and Parker, 2024). The found that DNA providers operate under a “zero-trust” model–i.e., treating every purchase as potentially dangerous until proven otherwise through thorough customer and sequence screening (Kane and Parker, 2024) – but that sensitivity of the screening method, monitoring and evaluation practices, screening pipelines, and screening approaches remained variable across companies (Kane and Parker, 2024). They attributed this heterogeneity to a lack of standardized oversight (Kane and Parker, 2024). More recently, in October 2025, researchers from a diverse team that included scientists from Microsoft, Raytheon BBN Technologies, IDT, Twist Bioscience, Battelle, Aclid, and IBBIS demonstrated that artificial intelligence could facilitate protein design strategies that could evade some synthesis screening tools (Wittmann et al., 2025). However, this red teaming effort also helped develop a software patch that flagged all but 3% of malicious sequence orders in a second attempt (Wittmann et al., 2025; Hutson, 2025).

Pursuant to their tasking in Section 4.4(a) (ii) of the October 2023 Executive Order (U.S. Department of Health and Human Services, Administration for Strategic Preparedness and Response, 2023), NIST had undertaken a process, in coordination with the Engineering Biology Research Consortium, to gather stakeholder feedback about how conformity with the sequence screening requirements could be verified and what measures could be taken to ensure uniform implementation of sequence and customer screening requirements (Engineering Biology Research Consortium (EBRC), 2026). Their 2025 report also included a draft NIST Standard Guide for Providers to support compliance with the 2024 NSTC Framework for Nucleic Acid Synthesis Screening (National Science and Technology Council, 2024). In addition, consistent with one of the recommedations in the EBRC report, the Johns Hopkins Center for Health Security had established an website that provided an explanation of how nucleic acid synthesis providers could comply with the provisions in the NSTC framework and that also housed a list of providers (and of manufacutures of benchtop nucleic acid synthesis devices) that had attested to their compliance. The purpose of this website was to aid federally-funded researchers in ensuring that their purchases of those materials met the requirements established by their funding agencies (Gene Synthesis Screening Information Hub, 2026). No formal methodology was established during this period to verify compliance with the NSTC framework, though the U.S. Department of Homeland Security had been tasked in the October 2023 Executive Order with developing structured evalution and stress testing mechanisms (U.S. Department of Health and Human Services, Administration for Strategic Preparedness and Response, 2023).

A working group had been organized under the IGSC to determine whether a genetic sequence test set could be established to support the harmonization of synthesis screening by IGSC members. This working group published a roadmap for creating such test data sets in mid-2024 (Wheeler et al., 2024). They noted that complexities introduced both by screening tool methodologies and by the lack of clarity from governments regarding which sequences to flag, and under what conditions–including clear guidance about how to treat sequences encoding proteins with unknown functions–make the uniform implementation of screening protocols difficult. They concluded by recommending that a test set be developed, based on the IGSC Regulated Pathogen Database, and noted that additional work would need to be done to meet the goal of expanding the test set beyond sequences from regulated agents. To address the uniform appreciation of these seqeunces and to support the establishment of robust test data sets, the IGSC Test Set Working Group–which had authored that report–was institutionalized as the Sequence Biosecurity Risk Consortium (SBRC), in 2024 (Engineering Biology Research Consortium (EBRC), 2026). To date, while SBRC leadership has published a roadmap toward the indistry-led development of a unified understanding of SOCs–both from regulated and non-gulated agents (Beal and Alexanian, 2025) – the SBRC has not issued test sets or standards.

## Changing course and potentially extending beyond research funding requirements in the United States

In May of 2025 the Trump Administration issued Executive Order 14292, which among other things rescinded the 2024 NSTC framework that would have established sequence and customer screening as a funding requirement for federally funded researchers (Executive Office of the President, 2025a). In rescinding it, the order directed the federal government to issue a revised set of funding requirements by August 2025. Furthermore, the Order tasked the development, by November 2025, of a strategy to extend sequence and customer screening requirements beyond federally funded research. Neither the revised policy nor the strategy to extend requirements beyond federally funded research have emerged as of the date of this publication, though the July 2025 release of “America’s AI Action Plan” by the Administration did indicate that nucleic acid synthesis and customer screening remained priorities for the Administration (Executive Office of the President, 2025c). In the absence of clarification about funding requirements and potential changes to previously issued guidance, the authors have gathered from personal communications with researchers that there is currently some confusion among U.S. academic researchers about which guidelines currently apply. For instance, the 2024 NSTC framework–which superseded, but did not explicitly rescind, the 2023 HHS recommendations–has itself been rescinded by EO 14292. This sequence of events would appear to leave the 2023 HHS recommendations still in effect. But some researchers have communicated with us that they interpreted the NSTC framework as rescinding the 2023 HHS recommendations by establishing federal life sciences funding requirements that parallelled those recommendations. There is a *de facto* lacuna in screening policies in the United States, and this type of uncertainty can be deleterious to the vitality of a research enterprise.

In January 2026, the Biosecurity Modernization and Innovation Act was introduced in the United States Senate (Biosecurity Modernization and Innovation Act, S. 3741, 119th Cong, 2026). This bill would require all companies selling synthetic nucleic acids or benchtop synthesis devices in the United States to screen for SOCs and to verify the legitimacy of customers ordering them. Notably, it also would require the Department of Commerce to establish a list of SOCs, with special attention to “sequences capable of creating pathogens with pandemic potential.” The importance of understanding which sequences are involved in endowing pathogenicity and toxicity to infectious agents, and decisions involved in the regulation, prohibition, or oversight of certain life sciences research that has high potential for misuse or that involves creating or enhancing potential pandemic pathogens, has been noted previously (Godbold et al., 2023) and is the focus of another article in this issue (Godbold et al., 2026).

## Paths forward

To remain apace with emerging capabilities, there is a need to ensure that sequences posing risks of causing harm to people are recognized as such uniformly across the life sciences research ecosystem, and that they are only provided to individuals who have a legitimate need for them. However, this should be done in such a way that does not unduly infringe upon the tremendous potential for societal good that has and continues to result from the appropriate use of these genetic sequences in biomedical research. The USG has undertaken the regulation of biological materials that pose significant risks to the public and to national security in the past, through the Federal Select Agent Program, and each of the last two presidential administrations has called for mechanisms to ensure that genetic sequence screening of synthetic nucleic acids is implemented uniformly in the United States.

The status of the policy development process tasked to federal agencies in the May 2025 by EO 14292 is unclear, and no policies have emerged as of this writing (more than 6 months after the policies were to have been released and 3 months after a strategy to extend screening requirements to non-federally funded research in the United States was due). Given that NIH is currently engaged in a year-long review of its biosafety and biosecurity policies (U.S. Department of Health and Human Services, 2025), it may be that the calls for sequence and customer screening as a funding requirement will be fulfilled in the coming year as part of revised NIH policies rather than ones issued by the White House. While some life sciences research is funded by other federal agencies that would not formally be bound by NIH-specific policies, a requirement established by NIH would likely result in almost all companies meeting the standards that they set, as NIH disburses most federal funding for life sciences research in the United States (National Science Foundation, 2026; U.S. Department of Health and Human Services, 2026).

Schools of thought about the urgency with which robust nucleic acid synthesis screening policies are needed seem to have become established based on different understandings of the risk of misuse that these materials pose and the need for regulations to ensure a healthy gene synthesis industry to support biomedical advancements.^16^ The low-risk perspective has advocated that screening requirements are largely performative, arguing that existing institutional oversight requirements–such as those accompanying NIH funding–have already established safe and secure practices in the research environments where synthetic genetic materials are being used. A moderate-risk perspective views screening as a vital mechanism for protecting the health of the gene synthesis industry–which has a critical support role for the broader bioeconomy–and that by standardizing due diligence, the industry benefits from liability protections. This incentivizes investment into these critical components and benefits biomedical research at large. Finally, the high-risk perspective warns of potential catastrophic outcomes, emphasizing that the democratization of biotechnology—enabled by AI-assisted instruction and design–means that a malign actor could engineer pandemic threats, necessitating universal and sophisticated screening.

Impressions about urgency with which harmonized industry practices or regulatory structures are needed vary among well-meaning biosecurity experts and academic researchers. The need to balance the remarkable potential of emerging synthetic biotechnologies for biomedical advancements and emerging technologies in fields not traditionally thought of as biotechnologies with the seemingly accelerating magnitude of the risks that synthetic genetic materials may pose are based upon assumptions about who could misuse biotechnologies and how they could do so. With anticipated near-term growth in the scope of biotechnological applications across the bioeconomy (Manyika et al., 2020), it is likely that the pool of individuals with the skills to misuse synthetic nucleic acids will similarly grow. Considering that large-language models also seem to have the potential to substantially lower the expertise barrier for misusing genetic materials (Brady et al., 2026), it seems prudent to exercise caution about the sale and distribution of these materials. Regulation should not be seen as antithetical to innovation–for instance the pharmaceutical industry has continued to benefit from innovation even in light of the current regulatory structures overseeing them (U.S. Department of Health and Human Services, 2024b). All industry players are well aware of the chilling effect that a disaster attributed to the use of synthetic DNA would have on their future markets, above and beyond the direct morbidity and mortality consequences. At the same time, avoiding burdening the U.S. research enterprise and not putting U.S. bioeconomic actors at an international disadvantage is also critical.

Finding the right balance between federal funding requirements, harmonized industry practices, and regulatory frameworks will likely require public private partnerships to establish best practices–by all entities involved in the provision, use, and transfer of these materials–that would guide those mechanisms. This article is not meant to prescribe a path forward, but to describe the milestones and guideposts that have shaped the evolution of policy in this area and that frame the critical elements of the next steps on that path.

## Footnotes

1 Several protein toxins are included in the Federal Select Agent Program’s Biological Select Agents and Toxins List, including ricin, abrin, and botulinum toxins, the conotoxins, and the staphylococcal enterotoxins.

2 Select agent viruses whose genomes are encoded by positive-sense and single-stranded RNA (+ssRNA).

3 Those select agent toxins, listed in footnote 1, which are proteins encoded by single genes.

4 In 2008, Craic Computing made Blackwatch open source to any DNA synthesis company that wanted it.

5 This legislation appears in the United States Code as 18 USC § 175c and includes the following definition. “‘variola virus’ means a virus that can cause human smallpox or any derivative of the variola major virus that contains more than 85 percent of the gene sequence of the variola major virus or the variola minor virus.”

6 Personal communication with John Mulligan, founder of Blue Heron Technologies. 17 April 2026.

7 Additional regulations and statutes govern the malicious use of biological agents, without a particular focus on synthetic nucleic acids.

8 One objection to a requirement that DNA synthesis firms screen their orders, as articulated by biotechnology analyst Rob Carlson, is that it would establish a perverse incentive to proliferate DNA synthesis capabilities in jurisdictions that had no such requirement. Such actions would create sources of unscreened DNA while undercutting firms that do screen. See https://static1.squarespace.com/static/57645a78ebbd1a1d25dcb511/t/669967004d20f00ee7d0a3b9/1721329409089/Carlson_NASEM_NSCEB_July_2024.pdf. However, nucleic acid vendors in the United States and Europe do not argue that screening requirements would have to be enacted globally to make them worthwhile.

9 The IGSC Harmonized Screening Protocol indicated that IGSC was assembling a Regulated Pathogen Database that would include data from “all organisms on the Select Agent list, the Australia Group List, and any other national list of regulated pathogens.” It also instructed companies that “[u]ntil this is deployed … [a]t a minimum, IGSC companies screen for all pathogen and toxin genes from the US Select Agents and Toxins List and/or from the list specified in paragraphs 1C351-1C354 of European Union Council Regulation 428/2009.”

10 Notably, crimes involving the use of weapons of mass destruction can be prosecuted under 18 USC § 2332a (Use of weapons of mass destruction). 18 USC § 3,286 (Extension of statute of limitation for certain terrorism offenses) establishes an 8-year statute of limitation for such crimes.

11 Upon introduction into a cell, viral genomes that consist of +ssRNA can be directly translated into viral proteins, which form virions within the cell, replicate the genomic material, and package those genomes into budding virions.

12 Personal communication with Sarah Carter, formerly of JCVI, and Anna Muldoon, formerly of ASPR. 17 April 2026.

13 The OSTP framework includes a requirement to report “potentially illegitimate purchase orders of synthetic nucleic acids involving SOCs or of benchtop nucleic acid synthesis equipment … to relevant authorities, including, where appropriate, to an FBI Field Office.”

14 The OSTP framework includes a requirement to retain “all screening records, including flagged orders; customer screening interactions, including when the orders were deemed acceptable; documentation of further action taken in response to flagged orders; and rationale for decisions about the legitimacy of customers whose orders were flagged, including where orders contained SOCs.”

15 The OSTP framework included a provision that records of orders may be retained to facilitate the identification of illegitimate orders that are split between Providers, in noting that “[r]etained records may help identify attempts to order components of SOCs from multiple entities for reassembly, and to support industry-wide collaborations to identify such orders,” and noted that “[s]uch efforts may become part of future adherence standards.”

16 The following discussion is drawn from a RAND report, titled Leveraging the Federal Select Agent Program to Oversee Nucleic Acids–Volume 2, Potential Benefits and Burdens, Recommendations, and Implementation Considerations., in preparation.
