# Correction: The evolution of the United States nucleic acid screening policies

**DOI:** 10.3389/fbioe.2026.1933412

**Published:** 2026-09-08

**Authors:** Gerald L. Epstein, Adeline E. Williams, Curtis Matthew Sharkey

**Affiliations:** RAND Corporation, Arlington, VA, United States

**Keywords:** biosecurity governance, dual use technologies, federal policy development, gene synthesis screening, synthetic biology

The following reference for Sharkey et al., 2024 was erroneously omitted from the reference list: Sharkey, C. M., Lekveishvili, M. L., de la Rosa, T., and Danskin, K. (2024). Enhancing gene synthesis security: an updated framework for synthetic nucleic acid screening and the responsible use of synthetic biological materials. *Appl. Biosaf*. 29 (2), 63–70. doi:10.1089/apb.2023.0036.

The reference for U.S. Department of Health and Human Services, 2023 was erroneously written as: Companion Guide to Assist (2023). *Companion Guide to Assist in Implementing the Recommendations of the Screening Framework Guidance for Providers and Users of Synthetic Nucleic Acids*. It should be: U.S. Department of Health and Human Services. (2023). *Companion Guide to Assist in Implementing the Recommendations of the Screening Framework Guidance for Providers and Users of Synthetic Nucleic Acids*. Washington, DC: Administration for Strategic Preparedness and Response. Available online at: https://aspr.hhs.gov/S3/Documents/SynNA-Companion-Guide-508.pdf.

The reference for U.S. Department of Health and Human Services, 2021a was erroneously written as: Double-Stranded DNA (2021). *Comments Received in Response to Federal Register Notice 2020-18444, Review and Revision of the Screening Framework Guidance for Providers of Synthetic Doublestranded DNA*. It should be: U.S. Department of Health and Human Services (2021a). *Comments Received in Response to Federal Register Notice 2020-18444, Review and Revision of the Screening Framework Guidance for Providers of Synthetic Double Stranded DNA*. Washington, DC: Administration for Strategic Preparedness and Response. Available online at: https://aspr.hhs.gov/S3/Documents/SynDNA-RFI-2020.pdf.

The reference for Gene Synthesis Screening Information Hub, 2026 was erroneously written as: Gene Synthesis Screening Information Hub (2026). *List of Framework-Attesting Nucleic Acid Synthesis Providers and Benchtop Manufacturers*. Johns Hopkins Center for Health Security. It should be: Gene Synthesis Screening Information Hub (2026). *List of Framework-Attesting Nucleic Acid Synthesis Providers and Benchtop Manufacturers*. Johns Hopkins Center for Health Security. Available online at: https://genesynthesisscreening.centerforhealthsecurity.org/for-customers/list-of-framework-attesting-providers-benchtop-manufacturers.

The reference for California State Assembly, 2022 was erroneously written as: Gene Synthesis Providers and Manufacturers (2022). *Gene Synthesis Providers and Manufacturers of Gene Synthesis Equipment A.B. 1963 (Cal. 2022). It should be: California State Assembly (2022)*. Assembly Bill No. 1963: Gene synthesis providers and manufacturers of gene synthesis equipment, Chapter 636. Available online at: https://legiscan.com/CA/text/AB1963/id/2603676.

The reference for U.S. Department of Health and Human Services, 2026 was erroneously written as: Grants and Funding (2026). Bethesda, Maryland: National Institutes of Health. It should be: U.S. Department of Health and Human Services. (2026). *Grants and Funding*. Washington, DC: National Institutes of Health. Available online at: https://www.nih.gov/grants-funding.

The reference for Gronvall, 2021 was erroneously written as: Hearing to Discuss HB1256 (2021). *Maryland Department of Health–Gene Synthesis Providers and Manufacturers of Gene Synthesis Equipment–Certification*. Testimony of Gigi Gronvall, PhD. It should be: Gronvall, G.K. (2021). *Testimony of Gigi Gronvall, PhD in Support of HB1256: Hearing to Discuss HB1256 (Maryland Department of Health–Gene Synthesis Providers and Manufacturers of Gene Synthesis Equipment–Certification)*. Maryland House Health and Government Operations Committee. Available online at: https://centerforhealthsecurity.org/our-work/testimonies-briefings/testimony-of-gigi-gronvall-phd-in-support-of-hb1256.

The reference for U.S. Department of Health and Human Services, 2021b was erroneously written as: *HHS Screening Framework Guidance for Providers and Users of Synthetic Oligonucleotides, Summary of Updates in Response to Public Comments Received in 2020*, (2021). It should be: U.S. Department of Health and Human Services (2021b). *Screening Framework Guidance for Providers and Users of Synthetic Oligonucleotides, Summary of Updates in Response to Public Comments Received in 2020*. Washington, DC: Administration for Strategic Preparedness and Response. Available online at: https://aspr.hhs.gov/S3/Documents/Summary-Updates-508.pdf.

The reference for Executive Office of the President, 2025a was erroneously written as: Improving the safety and security of biological research exec. *Order No. 14292*, (2025). It should be: Executive Office of the President (2025a). *Improving the safety and security of biological research. Executive Order 14292*. Available online at: https://www.whitehouse.gov/presidential-actions/2025/05/improving-the-safety-and-security-of-biological-research/.

The reference for U.S. Department of Health and Human Services, 2025 was erroneously written as: National Institutes of Health (2025). Statement: NIH launches initiative to modernize and strengthen biosafety oversight. In: *Services HaH*, editor.: National Institutes of Health. Available online at: https://www.nih.gov/about-nih/nih-director/statements/nih-launches-initiative-modernize-strengthen-biosafety-oversight. It should be: U.S. Department of Health and Human Services (2025). Statement: NIH launches initiative to modernize and strengthen biosafety oversight. Washington, DC: National Institutes of Health. Available online at: https://www.nih.gov/about-nih/nih-director/statements/nih-launches-initiative-modernize-strengthen-biosafety-oversight.

The reference for National Science and Technology Council, 2024 was erroneously written as: National Science and Technology Council (2024). Available online at: https://aspr.hhs.gov/S3/Documents/OSTP-Nucleic-Acid-Synthesis-Screening-Framework-Sep2024.pdf. It should be: National Science and Technology Council (2024). *Framework for Nucleic Acid Synthesis Screening*. Available online at: https://aspr.hhs.gov/S3/Documents/OSTP-Nucleic-Acid-Synthesis-Screening-Framework-Sep2024.pdf.

The reference for U.S. Department of Health and Human Services, 2024b was erroneously written as: NIH Guidelines for Research (2024b). *NIH Guidelines for Research Involving Recombinant or Synthetic Nucleic Acid Molecules*. It should be: U.S. Department of Health and Human Services (2024). *NIH Guidelines for Research Involving Recombinant or Synthetic Nucleic Acid Molecules*. Washington, DC: National Institutes of Health. Available online at: https://osp.od.nih.gov/wp-content/uploads/NIH_Guidelines.pdf.

The reference for U.S. Department of Health and Human Services, 2024a was erroneously written as: Novel Drug Advancing health through innovation: new drug therapy approvals 2024. (2024). In: *US Department of Health and Human Services FaDA, Center for Drug Evaluation and Research*, editor. Washington DC: FDA. It should be: U.S. Department of Health and Human Services (2024a). *Advancing Health Through Innovation: New Drug Therapy Approvals 2024*. Washington, DC: FDA, Center for Drug Evaluation and Research. Available online at: https://www.fda.gov/drugs/novel-drug-approvals-fda/novel-drug-approvals-2024.

The reference for Executive Office of the President, 2009 was erroneously written as: President, Executive Office (2009). Screening Framework Guidance for Providers of Synthetic Double-Stranded DNA 74 Fed. Reg. 62652. Executive Office of the President. Available online at: https://www.federalregister.gov/documents/2009/11/27/E9-28328/screening-framework-guidance-for-synthetic-double-stranded-dna-providers. It should be: Executive Office of the President (2009). *Screening Framework Guidance for Providers of Synthetic Double-Stranded DNA*. 74 Fed. Reg. 62652. Executive Office of the President. Available online at: https://www.federalregister.gov/documents/2009/11/27/E9-28328/screening-framework-guidance-for-synthetic-double-stranded-dna-providers.

The reference for Executive Office of the President, 2010 was erroneously written as: President, Executive Office (2010). Screening Framework Guidance for Providers of Synthetic Double-Stranded DNA 75 Fed. Reg. 62820. Washington, DC: Executive Office of the President. Available online at: https://www.federalregister.gov/documents/2010/10/13/2010-25728/screening-framework-guidance-for-providers-of-synthetic-double-stranded-dna. It should be: Executive Office of the President (2010). *Screening Framework Guidance for Providers of Synthetic Double-Stranded DNA*. 75 Fed. Reg. 62820. Washington, DC: Executive Office of the President. Available online at: https://www.federalregister.gov/documents/2010/10/13/2010-25728/screening-framework-guidance-for-providers-of-synthetic-double-stranded-dna.

The reference for European Commission, 2025 was erroneously written as: Proposal for a Regulation (2025). *Proposal for a Regulation of the European Parliament and of the Council on Establishing a Framework of Measures for Strengthening Union’s Biotechnology and Biomanufacturing Sectors Particularly in the Area of Health and Amending Regulations (EC) No 178/2002, (EC) No 1394/2007, (EU) No 536/2014, (EU) 2019/6, (EU) 2024/795 and (EU) 2024/1938 (European Biotech Act) COM(2025) 1022 Final*. It should be: European Commission (2025). *Proposal for a Regulation of the European Parliament and of the Council on establishing a framework of measures for strengthening Union’s biotechnology and biomanufacturing sectors particularly in the area of health and amending Regulations (EC) No 178/2002, (EC) No 1394/2007, (EU) No 536/2014, (EU) 2019/6, (EU) 2024/795 and (EU) 2024/1938 (European Biotech Act). COM(2025) 1022 final*. Brussels: European Commission.

The reference for Code of Federal Regulations, 2024a was erroneously written as: Possession, U. (2024a). *Transfer of Select Agents and Toxins 42 CFR Part 73*. It should be: Code of Federal Regulations (2024a). *Title 42 – Public Health: Part 73 – Select Agents and Toxins*. Available online at: https://www.ecfr.gov/current/title-42/chapter-I/subchapter-F/part-73.

The reference for Code of Federal Regulations, 2024b was erroneously written as: Possession, U. (2024b). *Transfer of Select Agents and Toxins 7 CFR Part 331*. It should be: Code of Federal Regulations (2024b). *Title 7 – Agriculture: Part 331 – Possession, Use, and Transfer of Select Agents and Toxins*. Available online at: https://www.ecfr.gov/current/title-7/subtitle-B/chapter-III/part-331.

The reference for U.S. Code, 2004 was erroneously written as: *Prohibitions with Respect to Variola Virus 18 U.S.C. § 175c*, (2004). It should be: U.S. Code (2004). *Title 18 – Crimes and Criminal Procedure: Part 175c–Variola Virus*. Available online at: https://www.govregs.com/uscode/title18_partI_chapter10_section175c.

The reference for Executive Office of the President, 2025b was erroneously written as: Removing Barriers to American Leadership (2025b). *Removing Barriers to American Leadership in Artificial Intelligence Exec*. Order No. 14179. It should be: Executive Office of the President (2025). *Removing Barriers to American Leadership in Artificial Intelligence*. Exec. Order No. 14179. Washington, DC. Available online at: https://www.whitehouse.gov/presidential-actions/2025/01/removing-barriers-to-american-leadership-in-artificial-intelligence/.

The reference for U.S. Department of Health and Human Services, 2022 was erroneously written as: Responses to Federal Register Notice (2022). *Screening Framework Guidance for Providers and Users of Synthetic Oligonucleotides*. It should be: U.S. Department of Health and Human Services (2022). *Responses to Federal Register Notice Screening Framework Guidance for Providers and Users of Synthetic Oligonucleotides*. Washington, DC: Administration for Strategic Preparedness and Response. Available online at: https://aspr.hhs.gov/S3/Documents/2022-SynOligo-FRN-All-Responses-508.pdf.

The reference for Biosecurity Modernization and Innovation Act was erroneously written as: S.3741 (2026). *S.3741 - Biosecurity Modernization and Innovation Act of 2026, (Introduced 2026)*. It should be: Biosecurity Modernization and Innovation Act, S. 3741, 119th Cong. (2026). Available at: https://www.congress.gov/bill/119th-congress/senate-bill/3741/text.

The reference for Executive Office of the President, 2023 was erroneously written as: Safe, S. (2023). *Trustworthy Development and Use of Artificial Intelligence Exec*. Order No. 14110. Available online at: https://www.federalregister.gov/documents/2023/11/01/2023-24283/safe-secure-and-trustworthy-development-and-use-of-artificial-intelligence. It should be: Executive Office of the President (2023). *Trustworthy Development and Use of Artificial Intelligence* Exec. Order No. 14110. Available online at: https://www.federalregister.gov/documents/2023/11/01/2023-24283/safe-secure-and-trustworthy-development-and-use-of-artificial-intelligence.

The reference for Department for Science, Innovation and Technology, 2024 was erroneously written as: Science, I. T. (2024). *UK Screening Guidance on Synthetic Nucleic Acids for Users and Providers*. It should be: Department for Science, Innovation and Technology (2024) *UK screening guidance on synthetic nucleic acids for users and providers*. Available online at: https://www.gov.uk/government/publications/uk-screening-guidance-on-synthetic-nucleic-acids.

The reference for U.S. Department of Health and Human Services, 2020 was erroneously written as: Services, U.S. Department of Health and Human (2020). Review and Revision of the Screening Framework Guidance for Providers of Synthetic Double-Stranded DNA 85 Fed. *Reg*. 51761. Available online at: https://www.federalregister.gov/documents/2020/08/26/2020-18444/review-and-revision-of-the-screening-framework-guidance-for-providers-of-synthetic-double-stranded. It should be: U.S. Department of Health and Human Services (2020). *Review and Revision of the Screening Framework Guidance for Providers of Synthetic Double-Stranded DNA*. 85 Fed. Reg. 51761. Available online at: https://www.federalregister.gov/documents/2020/08/26/2020-18444/review-and-revision-of-the-screening-framework-guidance-for-providers-of-synthetic-double-stranded.

The reference for National Science Foundation, 2026 was erroneously written as: Universities Report 8.1% Growth in R&D Expenditures in FY 2024, Reaching Over $117 Billion (2026). *National Center for Science and Engineering Statistics (NCSES)*. Alexandria, VA: National Science Foundation. It should be: National Science Foundation (2026). *Universities Report 8.1% Growth in R&D Expenditures in FY 2024, Reaching Over $117 Billion*. Alexandria, VA: National Center for Science and Engineering Statistics (NCSES). Available online at: https://ncses.nsf.gov/pubs/nsf26305.

The reference for Executive Office of the President, 2025 was erroneously written as: Winning the Race: America’s AI Action Plan, (2025). Available online at: https://www.whitehouse.gov/wp-content/uploads/2025/07/Americas-AI-Action-Plan.pdf. It should be: Executive Office of the President (2025). *Winning the Race: America’s AI Action Plan*. Available online at: https://www.whitehouse.gov/wp-content/uploads/2025/07/Americas-AI-Action-Plan.pdf.

The reference Nature, 2006 was erroneously written as: Policing ourselves (2006). *Policing ourselves*. Nat. 441 (7092):383–383. doi:10.1038/441383a. It should be: Nature (2006). Policing ourselves. Nat. 441 (7092):383. doi: 10.1038/441383a.

In the section **There will be a test**, paragraph 3, the citation for Engineering Biology Research Consortium (EBRC), 2025 was erroneously written as Engineering Biology Research Consortium (EBRC), 2026.

In the **Abstract**, an Executive Order was erroneously listed as number 14,292. This has been corrected to read:

“These were countermanded by Executive Order 14292 in mid-2025, leaving the path forward for gene synthesis screening uncertain.”

A correction has been made to the section **Industry consortium guidelines**, “Formation of the International Gene Synthesis Consortium”, paragraph 4. The word “companies” was erroneously included after “IGSC”. The corrected sentence is:

“IGSC also asked their member companies to keep written records of sequence and customer screening for 8 years”.

A correction has been made to the section **U.S. government guidance**, “Initial reaction to the guidance”, paragraph 2. The word “continue” was used instead of “continues”. The corrected sentence is:

“Some in the biosecurity community, as well as in industry, embraced the guidance, although the perception of the real risks from access to synthetic DNA differed, and continues to differ, to this day.”

A correction has been made to the section **Final updated screening guidance for the United States**, paragraph 1. The reference “Screening Framework Guidance for Providers, 2022” was erroneously included. The corrected sentence is:

“On 29 April 2022, ASPR published a draft revised screening framework guidance as a Notice in the Federal Register (87 FR 25495), which represented a significant expansion from the original 2010 guidance.”

A correction has been made to the section **From guidance to funding requirements–presidential action**, paragraph 7. An Executive Order was erroneously listed as number 14,110. This has been corrected to read:

“It also tasked the DHS with developing the structured evaluation and stress testing mechanisms called for in Executive Order 14110.”

A correction has been made to the section **There will be a test**, paragraph 2. The word “be” was missing after “could”. The corrected sentence is:

“Pursuant to their tasking in Section 4.4(a) (ii) of the October 2023 Executive Order (U.S. Department of Health and Human Services, Administration for Strategic Preparedness and Response, 2023), NIST had undertaken a process, in coordination with the Engineering Biology Research Consortium, to gather stakeholder feedback about how conformity with the sequence screening requirements could be verified and what measures could be taken to ensure uniform implementation of sequence and customer screening requirements (Engineering Biology Research Consortium (EBRC), 2025).”

A correction has been made to the section C**hanging course and potentially extending beyond research funding requirements in the United States**, paragraph 1. An Executive Order was erroneously listed as number 14,292. This has been corrected to read:

“In May of 2025 the Trump Administration issued Executive Order 14292, which among other things rescinded the 2024 NSTC framework that would have established sequence and customer screening as a funding requirement for federally funded researchers (Improving the Safety and Security, 2025).”

A correction has been made to the section **Paths forward**, paragraph 3. A space was missing. The corrected sentence is:

“Schools of thought about the urgency with which robust nucleic acid synthesis screening policies are needed seem to have become established based on different understandings of the risk of misuse that these materials pose and the need for regulations to ensure a healthy gene synthesis industry to support biomedical advancements.”

A correction has been made to the section **Paths forward**, paragraph 3. “Requirements” and “industry” were misspelled. The corrected sentences are:

“The low-risk perspective has advocated that screening requirements are largely performative, arguing that existing institutional oversight requirements–such as those accompanying NIH funding–have already established safe and secure practices in the research environments where synthetic genetic materials are being used. A moderate-risk perspective views screening as a vital mechanism for protecting the health of the gene synthesis industry–which has a critical support role for the broader bioeconomy–and that by standardizing due diligence, the industry benefits from liability protections.”

A correction has been made to **Footnote 16**. The title of a prepublication report that it references was incorrect. The correct footnote is:

“16 The following discussion is drawn from a RAND report, titled Leveraging the Federal Select Agent Program to Oversee Nucleic Acids–Volume 2, Potential Benefits and Burdens, Recommendations, and Implementation Considerations., in preparation.”

The original version of this article has been updated.

